# Multimodal feedback enhances human belief updating performance and reduces cognitive load in urban drone operation

**DOI:** 10.3389/frobt.2026.1707022

**Published:** 2026-06-10

**Authors:** Bowen Sun, Jiahao Wu, Hengxu You, Jing Du

**Affiliations:** Informatics, Cobots and Intelligent Construction (ICIC) Lab, Engineering School of Sustainable Infrastructure and Environment, University of Florida, Gainesville, FL, United States

**Keywords:** autonomous drones, haptic feedback, human belief update, human drone interaction, virtual reality

## Abstract

Supervisory control of autonomous drones in cluttered urban environments requires operators to update beliefs about dynamic hazards, such as localized wind changes, from imperfect and time-varying cues. To examine how interface design shapes this belief-updating process, four feedback conditions were compared: a baseline information panel (Control), augmented visual cues (Visual), upper-body haptic cues (Haptic), and their combination (Multimodal). Thirty participants supervised an autonomous drone in a Unity-based high-fidelity Virtual Reality simulation, with integrated eye tracking sampled at 90 Hz to derive an objective cognitive-load index. Belief updating was captured through change reporting and quantified using reaction latency and a Bayesian Beta-Binomial “local probability” metric that estimates time-resolved correctness, alongside subjective workload measured via NASA-TLX. Reaction latency decreased monotonically from Control (3.23 s) to Visual (2.61 s) to Haptic (1.67 s) to Multimodal (1.08 s). Multimodal was faster than Control (p < 0.001) and faster than Haptic (p = 0.045). Time-resolved correctness similarly improved, with mean local probability rising from 0.17 (Control) to 0.24 (Visual) and 0.22 (Haptic), reaching 0.43 under Multimodal. Eye-tracking comparisons indicated higher cognitive load in Haptic relative to Visual (p = 0.0201) and Multimodal (p = 0.0026). Together, the findings indicate a multimodal synergy that improves both speed and reliability of belief updates without the cognitive-load elevation observed under haptic-only feedback, supporting multimodal interface design for safer and more dependable human–autonomy teaming in urban drone operations.

## 1 Introduction

Unmanned aerial vehicles (UAVs), or drones, used in complex urban operations such as search and rescue, logistics, and surveillance have introduced new paradigms for human–robot interaction (HRI) (Lyu et al., 2023; Tezza and Andujar, 2019). While this technological advancement offers enhanced efficiency, the operation of drones in dense urban environments presents formidable challenges. These landscapes are cluttered with both static obstacles, like buildings and power lines, and dynamic elements, including vehicles and pedestrians, which complicate navigation and mission execution for autonomous systems (Gugan and Haque, 2023; Watkins et al., 2020). In practice, these complexities can exceed what onboard autonomy can resolve reliably in real time, shifting part of state assessment and risk management to supervisory control where the human must interpret system outputs and intervene when needed (Sheridan, 1992; Parasuraman et al., 2000).

A central challenge in HRI, particularly in supervisory control roles, is that both the human operator and the intelligent agent must often form critical assessments of the environment based on partial and imperfect information (Cai et al., 2022). The accuracy of these initial beliefs is paramount, as errors can have severe consequences. In a search and rescue mission, for example, a flawed assessment can delay the location of individuals in distress. Similarly, in logistics, inaccurate environmental reads can lead to mission failure and economic loss (Engesser et al., 2023; Lyu et al., 2023). The core difficulty lies in accurately estimating environmental states, such as localized wind conditions, from the limited sensor data provided by the drone (Watkins et al., 2020; Cai et al., 2022). In dense urban environments, however, wind is highly localized because surrounding buildings, street canyons, and corners can redirect and intensify airflow, causing the wind acting on the drone to differ substantially from the nominal background condition. Without timely and reliable estimation of these localized wind conditions, the drone may drift off its intended path, lose obstacle clearance, or require delayed human intervention, making localized wind estimation a practically important test case for studying belief updating under partial observability.

For the human supervisor, this task is further compounded by inherent cognitive limitations. The process of interpreting continuous, multimodal data streams can induce cognitive overload, a state where the volume of information exceeds an individual’s processing capacity, leading to degraded judgment (Dror, 2020; Rutkowski and Saunders, 2018; Sweller, 1988). This is especially pertinent in high-stakes drone operations that demand simultaneous monitoring of flight dynamics, environmental data, and mission objectives under time pressure (Liu et al., 2017; Wu et al., 2025). Furthermore, individual cognitive biases can distort an operator’s interpretation of data, leading to inaccurate belief formation (Dror, 2020). These concerns are consistent with prior human factors research showing that supervisory control in dynamic systems depends on maintaining situation awareness and allocating limited cognitive resources across concurrent tasks, both of which can degrade under high workload (Endsley, 1995).

Traditional cognitive models may not adequately capture the rapid, dynamic decision-making required in modern HRI scenarios. A deeper understanding of how humans process information from autonomous systems is needed to design interfaces that are more intuitive and effective. Because evidence in supervisory control arrives sequentially and with uncertainty, the process of forming a mental model can be formalized as a probabilistic inference problem rather than a purely categorical or static judgment. Bayesian belief updating provides a principled framework for describing how beliefs should change as new evidence is observed, where beliefs are adjusted in proportion to how informative new information is relative to existing knowledge (Knill and Pouget, 2004). This theoretical perspective clarifies why interface design is not merely a usability layer; rather, interface choices shape the evidence stream available to the human (e.g., salience, timing, and ambiguity) and therefore can influence both the speed and reliability of belief revision. In typical operations, the visual channel is often heavily loaded and cannot always convey latent forces like wind without adding attentional demand; conversely, the haptic channel can deliver time-critical cues with reduced dependence on continuous visual attention (Wickens, 2002; van Erp, 2007; Lutnyk et al., 2023). In workload terms, distributing information across complementary sensory channels may alter resource competition and mitigate overload risk during multitask monitoring (Wickens, 2002). Accordingly, feedback modality is conceptualized as a controlled manipulation of the evidence stream for detecting wind changes, enabling testable hypotheses that more salient and timely cues will reduce belief-update latency and increase time-resolved correctness, while workload indices capture whether these gains incur additional cognitive demand.

This study aims to provide empirical evidence on how different sensory feedback modalities impact the crucial “perception to belief” phase of decision-making in drone supervision. Through a controlled experiment, this research examines how interfaces incorporating augmented visual and haptic feedback affect an operator’s decision accuracy, belief updating, and cognitive load. The design targets a core supervisory task: detecting and responding to change in an environment variable (e.g., wind) when only partial, noisy evidence is available through the autonomy pipeline. In this context, belief updating refers to the operator reporting that the environmental wind has changed based on the presented information—such as realizing the wind has changed intensity or direction—rather than providing a quantitative estimation of specific values.

The remainder of this paper will review relevant literature in HRI and cognitive psychology, detail the experimental system design and methodology, and present an analysis of the results. Finally, it will discuss the findings, acknowledge limitations, and propose directions for future research. The overarching goal is to advance the design of HRI systems that foster safer and more efficient human–drone teaming in complex operational environments.

## 2 Related work

### 2.1 Human-robot interaction in drone operations

As drones have become more prevalent across domains such as logistics, inspection, and search and rescue, the study of human–drone interaction has emerged as a distinct area of inquiry within HRI. While early drones relied on complex control interfaces suited only for highly trained pilots, advances in automation have shifted the operator’s role from direct control to supervisory decision-making (Albeaino et al., 2022; Di Vincenzo et al., 2022; Tezza and Andujar, 2019). This transition is increasingly visible across the breadth of HDI use cases documented in recent syntheses of the field, which highlight the growing diversity of operational roles and interaction demands.

Unlike many ground-based or subsea-based HRI scenarios, human-drone interaction introduces constraints tied to flight in three-dimensional space, physical separation between human and robot, high volume data transmission with noise and artifacts removal and rapid shifts in viewpoint and context. These characteristics can fundamentally change how humans perceive, anticipate, and control robot behavior, and they motivate treating HDI as more than a direct transfer of interaction assumptions from ground robotics.

With advancements in automation and intelligent technologies, drones have begun to possess more complex navigation and task execution capabilities, and human-drone interaction has become increasingly important at this stage (Albeaino et al., 2022; Tezza and Andujar, 2019). In recent decades, research on user interfaces has become very popular. It has evolved from the earliest command-line interfaces to graphical control interfaces, and more recently to natural user interfaces (NUIs) (Suárez Fernández et al., 2016). NUIs for drones are a type of user interface that allows users to interact with drones through body movements, gestures, voice, and even real-world interactions, enabling operators to handle virtual or real entities more intuitively and realistically (Di Vincenzo et al., 2022; Tezza and Andujar, 2019).

Technologies like Augmented Reality (AR) and Virtual Reality (VR), part of Natural User Interfaces (NUI), enhance human-computer interaction through immersive experiences (Erat et al., 2018; Postal et al., 2016). Some operator-centric drone control systems utilize AR and VR to provide augmented views to human operators, allowing them to control the direction of the drone’s flight by gazing at it, thus enabling natural human-drone interaction (Büttner et al., 2020; Erat et al., 2018). There has also been research to develop a voice controller that recognizes operator voice commands to control fixed-wing semi-autonomous drones (Quigley et al., 2004). Wu and Sun designed a system capable of providing haptic feedback to drone operators, and the results show that haptic feedback manipulation can positively impact drone operators with previous drone flight experience, and that multimodal interactions can spread the sensory workload to enhance situational awareness, reduce cognitive load, and improve task accuracy (Sun et al., 2025; Wu et al., 2025). Carine Rognon and colleagues developed a soft haptic device that simulates the sensations experienced by operators during drone flight, addressing the challenge of rendering centripetal force relative to the drone on the human remote operator’s body. This haptic feedback reduces mental, physical, and time workload, improving user situational awareness (Rognon et al., 2019; Rognon et al., 2018). Multimodal perception has also been applied to unmanned aircraft interaction, with some systems allowing users to control drones in indoor environments using different methods and switching between them (Suárez Fernández et al., 2016), research on identification recognition has also shown that different modalities will effect the performance of biometric identification system when information is partially observable (Saleem et al., 2020).

Collectively, prior work demonstrates a growing toolbox of interaction modalities for drone supervision. However, field-level reviews repeatedly note that HDI research still faces open issues regarding how interaction designs should be evaluated and compared across operational contexts, particularly when human factors and uncertainty play central roles. In many studies, evaluation emphasizes control usability, task success, or aggregate workload/awareness measures, leaving less clarity on how specific modalities shape the operator’s belief formation and belief revision under partial observability, such as whether faster or more intuitive cues reliably translate into more accurate and timely belief updates when critical environmental states (e.g., wind) must be inferred rather than directly observed. This gap motivates examining the cognitive constraints and belief-updating mechanisms that link modality design to supervisory performance in uncertain environments, which is the focus of the next section.

### 2.2 Cognitive load in HRI

In supervisory control of autonomous drones, the operator’s role extends beyond issuing commands to continuously interpreting dynamic data streams and forming judgments about the environment. This process is inherently constrained by cognitive capacity (Endsley, 2017; Parasuraman et al., 2000). Consistent with foundational capacity accounts of attention and workload and cognitive load theory (Sweller, 1988). Cognitive load arises when the complexity, volume, or temporal pressure of incoming information exceeds the operator’s ability to process it effectively, which can slow responses and degrade decision quality (Endsley, 2017). Interfaces that present excessive, poorly structured, or poorly timed information can induce overload and reduce effective cue integration, including through attentional narrowing under stress or arousal (Easterbrook, 1959).

A critical cognitive mechanism underlying performance in such settings is belief updating, i.e., the process by which an operator revises their internal assessment of environmental states (e.g., wind conditions, obstacle positions, or risk levels) considering new but often incomplete or ambiguous information (Hogarth and Einhorn, 1992). Accurate and timely belief updating is essential for maintaining situational awareness and preventing errors in supervisory control. However, under high workload, operators may rely on heuristics or exhibit systematic biases that distort this process (Bourne and Yaroush, 2003; Hammond, 2000). For example, when cognitive resources are strained, operators may overweight immediately salient cues while neglecting others (Endsley, 2017), resulting in incomplete or delayed belief revisions. From a resource-competition perspective, these effects are also consistent with multiple resource theory, which predicts increased interference and cue neglect when concurrent demands draw on shared perceptual/cognitive resources (Wickens, 2002).

Existing HRI and human–automation workload research establishes robust links between interface demands, mental workload, and supervisory performance, but many evaluations emphasize aggregate outcomes (e.g., task success, overall workload, broad situation-awareness measures) rather than explicitly characterizing the time-resolved dynamics of belief revision that precede action (van de Merwe et al., 2024). In particular, there remains a need for task-aligned metrics that quantify belief-updating efficiency (speed and correctness) alongside workload, especially when evidence is distributed across different sensory channels that may change resource competition (Wickens, 2002). These needs motivate drawing on cognitive psychology models of belief updating to operationalize belief revision more explicitly and to formulate testable predictions about how interface modality shapes the evidence stream and resulting belief-updating behavior (Hogarth and Einhorn, 1992).

Aggregate outcomes such as task success are valuable, but they can be too coarse to reveal how an interface supports human supervision under uncertainty. In the present setting, two feedback conditions could produce similar final mission success because the autonomous controller may still complete the route, yet differ substantially in how quickly and reliably the operator detects a wind change, how much effort is required to interpret the cue, and how prepared the operator is to intervene before a near-failure develops. Explicitly modeling belief updates over time therefore provides additional insight into the mechanism linking interface design to supervisory performance: it distinguishes whether a modality improves the speed, reliability, and cognitive cost of human inference, rather than only the eventual task outcome.

### 2.3 Human belief update research

Human belief updating is a foundational topic in cognitive psychology addressing how individuals revise beliefs in response to new information (Hogarth and Einhorn, 1992). Research spans normative and descriptive perspectives, covering everyday judgment as well as high-stress decision contexts, and it documents systematic departures from idealized updating attributable to bounded cognition, heuristics, and bias processes (Hogarth and Einhorn, 1992).

One prominent normative framework is Bayesian updating, in which beliefs are revised in proportion to the evidential impact (i.e., informativeness or “surprise”) of new observations relative to prior beliefs (Bissiri et al., 2016; Jaffray, 1992). However, descriptive work shows that human belief revision frequently deviates from Bayesian prescriptions, particularly when reasoning is influenced by prior commitments, limited processing resources, or task framing (Nickerson, 1998; Trippas et al., 2015). For instance, confirmation-related effects where evidence consistent with existing beliefs is preferentially weighted and contradictory evidence is discounted are widely documented, and they can systematically distort updating even when new information is objectively diagnostic (Klayman, 1995; Nickerson, 1998).

Beyond bias tendencies, belief updating is sensitive to cognitive constraints. When information is complex, excessive, or arrives under time pressure, individuals may show reduced integration quality and less normatively consistent updates, consistent with accounts emphasizing limited cognitive resources and stress-related performance trade-offs (Bourne and Yaroush, 2003; Hammond, 2000; Kappes and Sharot, 2019). Individual differences further complicate belief revision: differences in reasoning strategies and working memory capacity have been linked to how people allocate attention to evidence and how resistant they are to belief-biased inferences (de Chantal et al., 2020; Trippas et al., 2015).

Although belief-updating research offers strong theoretical tools and measurable constructs, it is still uncommon for drone supervisory HRI studies to instantiate belief updating as a time-resolved, task-specific process under partial observability, despite repeated calls in HDI syntheses to improve evaluation practices and address human-factors challenges in real-world deployments (Herdel et al., 2022; Lingam et al., 2025; Tezza and Andujar, 2019). Similarly, while interface research explores new modalities (visual, haptic, multimodal), evaluation is often reported at the level of task success, usability, workload, or situation-awareness proxies rather than being grounded in an explicit belief-updating model that explains how and why a modality changes evidence integration (van de Merwe et al., 2024). This motivates integrating modality design treated as a controlled manipulation of the operator’s evidence stream with measurable belief-updating outcomes (speed and time-resolved correctness) and workload in a controlled drone-supervision task.

### 2.4 Summary of related works

In summary, prior human-drone interface research has expanded modality options (visual augmentation, haptics, multimodal interaction), and HRI human-factors research has established that cognitive load constrains supervisory performance. However, the literature rarely connects modality design to an explicit, measurable belief-updating process under partial observability. The present work addresses this gap by treating feedback modality as a controlled manipulation of the operator’s evidence stream and evaluating resulting belief-update speed and time-resolved correctness alongside cognitive-load measures. Table 1 shows the summary of the key related works.

**TABLE 1:** Summary of key related works.

Prior research stream	Key related works	Typical interfaces and settings	Typical outcomes reported	Key shortcomings related to this paper	How the present study addresses the gap
HDI shift from manual control to supervisory roles	Albeaino et al., 2022; Di Vincenzo et al., 2022; Tezza and Andujar, 2019; Parasuraman et al., 2000; Sheridan, 1992	Supervision of increasingly autonomous drones; operator monitoring and intervention	Role framing, interaction needs, high-level performance and usability considerations	Often does not operationalize belief revision as a measurable time-resolved process under partial observability	Defines a supervisory change-detection task (wind changes) and measures belief-update latency and time-resolved correctness under uncertainty
Natural user interfaces for drones (gesture/embodied interaction)	Suárez Fernández et al., 2016; Di Vincenzo et al., 2022; Tezza and Andujar, 2019	Gesture, body movement, gaze/embodied control metaphors	Intuitiveness, learnability, control usability, task success	Evaluation typically emphasizes control usability/task success rather than belief updating quality (speed vs. correctness)	Treats modality as an evidence-stream manipulation and evaluates belief updating (speed and correctness) alongside workload
AR/VR operator-centric supervision interfaces	Erat et al., 2018; Postal et al., 2016; Büttner et al., 2020	AR/VR displays, augmented views, immersive control/supervision	Usability, operator experience, task performance, SA proxies	Visual augmentation can increase visual-channel demands; limited modeling of how evidence presentation affects belief revision	Unity-based VR supervision with defined change events; compares visual vs. haptic vs. multimodal to test evidence salience/timing effects on belief updating and workload
Haptic/vibrotactile feedback for drone supervision and flight sensation rendering	Rognon et al., 2018; Rognon et al., 2019; Sun et al., 2025; Wu et al., 2025	Wearable haptics, vibrotactile cues, multimodal supervision	Workload (NASA-TLX), situational awareness, task accuracy, user experience	Often reports aggregate outcomes; limited time-resolved belief-update metrics that separate “fast” from “correct,” especially under partial observability	Adds belief-update latency + Bayesian Beta–Binomial detection probability and time-resolved correctness (“local probability”), enabling explicit speed–reliability analysis with workload trade-offs
Cognitive load and situation awareness in supervisory HRI	Endsley, 2017; Parasuraman et al., 2000; Endsley, 1995; Wickens, 2002	Supervisory monitoring under multitask/time pressure	Workload/SA frameworks; performance degradation under overload	Mechanism-level belief updating is discussed but rarely operationalized as a measurable, event-based process over time	Operationalizes belief updating as event-based and time-resolved metrics and directly links them to modality-driven evidence streams and workload measures (subjective + eye tracking)
Psychological models of belief updating (order effects, biases, bounded rationality)	Hogarth and Einhorn, 1992; Klayman, 1995; Trippas et al., 2015; Kappes and Sharot, 2019; Bourne and Yaroush, 2003; Hammond, 2000; de Chantal et al., 2020	Lab and applied judgment/decision tasks; varying stress and information complexity	Bias patterns, individual differences, deviations from normative updating	Rarely instantiated in drone supervisory tasks with controlled evidence delivery and multimodal interfaces; limited linkage to HRI workload/SA evaluation	Instantiates belief updating in a drone-supervision task and tests how modality (visual/haptic/multimodal) changes evidence integration, belief-update outcomes, and workload
Normative Bayesian updating foundations	Bissiri et al., 2016; Jaffray, 1992; Knill and Pouget, 2004	Bayesian/uncertainty frameworks for belief revision	Normative update rules; uncertainty handling	Normative models alone do not specify interface-level mechanisms or workload impacts in HRI	Uses Bayesian framing to define measurable belief-update reliability (Beta–Binomial) and tests interface modality effects on evidence salience/timing and cognitive cost in VR supervision

Beyond showing that modalities differ, this framework enables a more principled way to design and evaluate supervisory interfaces under uncertainty. Specifically, it allows researchers and practitioners to distinguish whether a feedback modality improves the speed of belief updating, the reliability of belief updating, or both, and whether such gains are achieved with additional cognitive cost. This distinction is useful for interface design because it can reveal when a modality appears effective based on task success alone but in fact imposes hidden workload or produces less stable human inference over time. More broadly, the framework supports the development of adaptive and human-centered supervisory systems by providing process-level metrics that can guide modality selection, multimodal cue integration, and future predictive models of operator behavior under partial observability.

## 3 Simulation and user interface design

### 3.1 Overview

**FIGURE 1:**
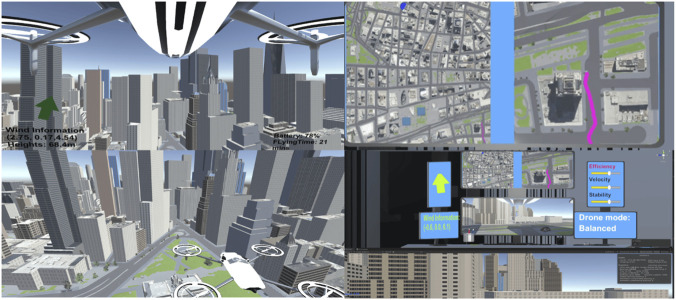
Simulator overview.

To examine how different sensory modalities affect human belief updating, this study designed a realistic simulation environment under a drone city search and rescue scenario in Unity (Figure 1). This human-drone sensory sharing system is designed to collect behavioral data and enhance human-drone interaction by providing a more intuitive and synchronized experience for operators. It consists of the autonomous drone control module, the augmented sensory feedback module, and the user interface module. The autonomous drone control module reads data from the urban environment, such as dynamic features of the wind fields, the drone’s current posture, obstacle distance, and target distance. It then employs a pretrained reinforcement learning (RL) model to control the drone in navigating complex urban terrains. The augmented sensory feedback module integrates multiple modalities to provide feedback to the human operator during the flight, including augmented visual feedback and haptic feedback. Specifically, the dynamic features of the winds are visualized in a head-mounted display (HMD) with particle and vector tools in Unity, including the wind direction and velocity. A haptic suit conveys tactile information to the human operator, allowing them to feel changes in wind conditions, ensuring an augmented sensory experience. The user interface module is designed for intuitive and efficient interaction between the human operator and the drone system, featuring multiple monitors that display critical information. It also allows the human operator to interrupt, change the drone’s priorities when needed. Each module of the proposed system plays a crucial role in enhancing the interaction between human operators and autonomous drones, creating a robust framework for assessing and improving drone performance in complex urban environments. No auditory wind cues were present in the simulation. The VR environment operated without wind-associated sounds, participants received no acoustic information about wind state from the simulation at any point during the experiment.

### 3.2 Simulation environment settings

To study human-autonomous drone interactions, this study developed a sophisticated simulator within the Unity engine, featuring a detailed model of a DJI Mavic 2 Pro. The core of the simulator is a custom-built landscape representing a 3,000-foot square area of Manhattan. While realistic textures from the Unity Asset Store were used for visual fidelity, this study manually constructed the primary building meshes. This approach was crucial, as it allowed us to create simplified colliders (bounding boxes) optimized for physics-based wind simulation. The street-level environment was intentionally simplified to isolate the core navigation challenge, including streets, sidewalks, and streetlights, but omitting dynamic objects like vehicles or pedestrians. Crucially, only the building meshes were equipped with colliders, defining them as the only physical obstacles in the flight path. The entire scene is illuminated by a fixed directional light set at a 45-degree angle to simulate consistent lighting conditions. Targets were placed on the map’s corners to create a complex flight route. A 200-foot altitude limit was imposed during training, forcing the drone to navigate through this custom-designed urban terrain.

To complement the static landscape, this study developed a dynamic wind simulation to create a robust and realistic training environment. The primary challenge was generating authentic aerodynamic scenarios without the prohibitive computational cost of traditional Computational Fluid Dynamics (CFD), which is unsuitable for the thousands of iterations required by Deep Reinforcement Learning (DRL). The solution utilizes a custom-built Convolutional Autoencoder, a representation model that is highly efficient at approximating complex airflow. While the detailed technical methodology for this model is discussed in previous studies (Sun et al., 2025; Wu et al., 2024), the process begins by defining an initial, global wind condition (speed and direction) across the entire landscape. At predefined intervals, this study systematically alter this global wind state. Table 2 shows the wind settings used across all conditions. The numbers in the base direction mean the angle between the wind and the map’s north directions. In experiment design, the main wind settings, including direction and magnitude, change at a random interval with a mean value of 20 s. Once a new state is set, the model takes this information, along with the fixed geometry of the Manhattan buildings, as inputs. It then rapidly calculates the resulting localized wind field, simulating the intricate ways airflow interacts with the urban architecture to create a detailed map of varying wind speeds and turbulence.

**TABLE 2:** The wind settings for the experiment.

Sequence	Base directions (°)	Base magnitude
1	90	0.3
2	270	0.6
3	0	0.2
4	180	0.8
5	90	0.5
6	180	0.4
7	0	0.8
8	270	0.9

Although these wind changes are consistent across all conditions, the wind’s impact on the drone varies significantly due to the drone’s position. Consequently, the drone in each condition receives different wind data throughout the flight, which helps to reduce the learning effect during the experiment. This methodology provides a highly realistic training environment by mimicking how a static city experiences changing weather, forcing the drone to learn a generalizable navigation policy rather than memorizing a single path. This entire approach is made feasible by the model’s computational efficiency, which generates a new wind field in seconds. In summary, this setup provides a controlled yet complex and dynamic testbed for developing and evaluating autonomous drone navigation systems.

### 3.3 Autonomous drone control module

The Autonomous Drone Control Module is the core intelligence of the system, designed to enable complex navigation through the challenging urban environment. The module is powered by a Deep Reinforcement Learning (DRL) agent trained using a Multi-Objective Reinforcement Learning (MORL) framework (Nguyen et al., 2020). This approach is essential for enabling the agent to balance multiple, often conflicting, flight objectives such as minimizing travel time, ensuring complete obstacle avoidance, and mitigating the effects of dynamic wind.

For the training algorithm, Proximal Policy Optimization (PPO) (Schulman et al., 2017) was employed due to its recognized stability, efficiency, and robustness in complex control tasks. It trains the agent by optimizing a policy, denoted as $\pi \left(a|s\right)$ ,which defines the probability of taking an action a in each given state s . The algorithm’s objective is to maximize the expected cumulative reward, which is formally defined as Equation 1:

$$\max_{\theta }E\left[\sum \sum_{t=0}^{T}\gamma^{t}r_{t}\right]$$ (1)

Where γ is the discount factor that balances immediate and future rewards, $r_{t}$ is the reward received at time step t , and θ represents the parameters of the neural network policy that are adjusted during training. To enhance the agent’s decision-making in dynamic environment, a Long Short-Term Memory (LSTM) network is integrated into the network (Graves, 2012; Hochreiter and Schmidhuber, 1997). The LSTM is critical, as it provides the agent with a memory of its recent actions and environmental states, allowing it to make informed decisions and learn from past failures, such as getting trapped in strong wind zones. The policy was implemented as a recurrent PPO network with two fully connected layers of 256 units each and one LSTM layer with a hidden size of 256. The shared feature encoder was followed by separate actor and critic output heads.

The agent’s decision-making is based on a comprehensive set of observations at any given moment when interacting with environment. All observations are derived from simulated built-in sensors to mirror real-world operational constraints. The network processes these inputs, and its output is a continuous action space, defining the precise thrust and directional control needed to navigate when acquire those observations. The inputs are organized into three main categories.

- Geometric Data: Information about the location and distance of the nearest buildings around the drone, captured via 3D ray sensor in Unity.
- Flight Trajectory: Critical flight data such as the drone’s velocity, its location, and the vector to its target.
- Wind Information: Data on the drone’s drift caused by wind forces and a memory of the last encountered strong wind zone.

Based on the observations, the MORL framework is guided by a carefully engineered reward function $R_{total}$ ​, which is a composite of several components designed to shape the drone’s behavior. The total reward is defined as Equation 2:

$$R_{total}=R_{distance}+R_{target}+R_{wind}+R_{coll}+R_{trap}+R_{timeout}+R_{time}$$ (2)

Each component serves a specific purpose in achieving the desired navigation policy. $R_{distance}$ ​ encourages the drone to make consistent progress toward its target. $R_{target}$ provides a large positive reward upon successfully reaching the destination. $R_{wind}$ ​ rewards the agent for successfully navigating through or around strong wind zones. $R_{coll}$ applies a significant penalty for any collision with buildings or other obstacles. $R_{trap}$ penalizes the drone for getting stuck in a local trap, particularly in strong crosswinds. $R_{timeout}$ imposes a penalty if the drone fails to reach the target within a set time limit. $R_{time}$ applies a small time-step penalty to encourage efficient path planning.

To develop a robust navigation policy, this study implemented a structured, multi-phase training curriculum. In the initial phase, the agent was trained in the Manhattan environment without any wind. The sole objective was to reach the designated targets and avoid collisions with static obstacles. This foundational training, conducted over approximately 80,000 epochs, established a baseline policy for basic navigation. In the second phase, this study introduced the dynamic wind fields generated by custom simulation model. The agent, building upon its initial policy, was forced to adapt to the aerodynamic forces pushing it off its intended course. The reward function was updated to penalize collisions caused by wind drift, encouraging the agent to learn counter-maneuvering strategies. The final and most advanced phase focused on teaching the agent to handle extreme conditions. This phase relied on the $R_{trap}$ and the penalty for lingering in strong winds to teach the agent these advanced detour strategies. This study also identified scenarios where the wind force in narrow corridors exceeded the drone’s thrust, causing it to become trapped which will be recorded as a “timeout” failure. Using a targeted retraining strategy, whenever a timeout occurred, the agent was repeatedly placed back into that challenging scenario. Thanks to its LSTM memory, the agent could recall the sequence of actions that led to the failure. A significant penalty was associated with lingering in these strong wind zones, incentivizing the agent to abandon its direct path and learn complex detour strategies. After over 100,000 total training epochs, the final agent demonstrated the ability to intelligently reroute around invisible, high-risk aerodynamic zones, ensuring both mission success and safety in a complex, dynamic urban landscape.

### 3.4 Perception sharing module

Building on previous research on multimodal feedback systems (Xia et al., 2024; Zhou et al., 2023; Zhu et al., 2022), this research developed the perception sharing system by incorporating information panels, augmented visual feedback and upper body haptic feedback together to establish a sensory-sharing pipeline for drones operating in complex urban environments. For augmented visual feedback, Participants interacted with the system through a VR headset that provided an immersive first-person drone view and integrated eye-tracking sensors (90 Hz). For haptic feedback, this study chose the upper body because tactile sensitivity is markedly higher in the upper body, enabling more precise and effective feedback. Additionally, prioritizing the upper limbs minimizes disturbance with drone operation. Since human primarily rely on the hands and forearms to manipulate controllers, the system deliberately avoids these regions. Although the drones in this study fly autonomously, operators still need to use their hands to set flight modes and report environmental changes, so their hand movements should not be impeded.

**FIGURE 2:**
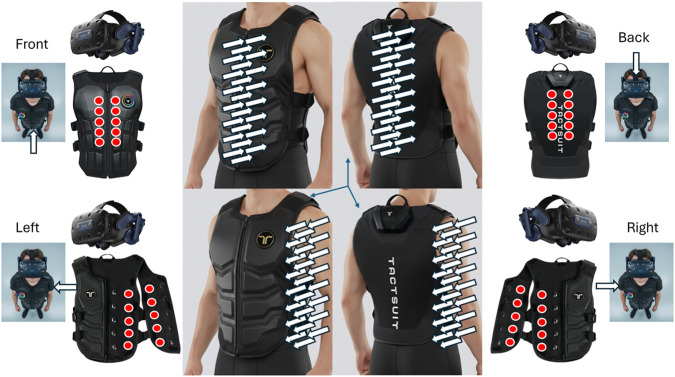
Perception sharing system with upper body haptic suit and VR headset.

Notably, the upper body haptic augmentation system shown in Figure 2 use bHaptic Suit to deliver real-time tactile cues about the environment surrounding the autonomous drone, with a focus on wind information that is essential for safe and efficient navigation. An array of vibration motors embedded in the suit can react to wind direction and speed through spatial activation patterns and intensity modulation, the output of each motor varies with wind magnitude: gentle vibrations indicate low wind speed, whereas stronger vibrations correspond to higher wind speed. To align device output with dynamic wind conditions, wind speed is mapped to a [0,1] force factor based on its ratio to the drone’s maximum speed Equation 3. When wind speed exceeds the drone’s capability, the operator receives feedback at maximum intensity. Figure 2 presents four typical haptic patterns designed for the principal wind directions.

$$Force\;factor=\min\left(1,\frac{\left|\left|v_{wind}-v_{drone}\right|\right|}{v_{drone_{m\text{ax}\;}}}\right)$$ (3)

The suit integrates 20 vibration motors on each side, and feedback is spatially differentiated by wind direction: wind from the right activates actuators on the right panel to simulate lateral pressure; wind from the rear, left, and front respectively triggers the motors on the back, left, and front panels. For an arbitrary wind direction, this study decompose it into two orthogonal components: first generate the patterns for the two perpendicular winds and then superimpose them, providing immediate, intuitive cues that help operators quickly interpret the environment and respond. In this study, the haptic feedback was implemented as a continuous state-based cue rather than an event-only alert. That is, whenever wind was present, the suit continuously encoded the current local wind direction through spatial activation and the current wind magnitude through vibration intensity, with the pattern updating as the wind field changed over time. Wind changes were therefore perceived through changes in actuator location and/or vibration strength rather than through a separate discrete warning signal. No formal discomfort or fatigue questionnaire specific to persistent vibration was administered; however, participants completed the experimental sessions without reporting major issues that required stopping or modifying the haptic condition. We acknowledge that prolonged exposure to continuous haptic stimulation may introduce usability concerns such as adaptation, discomfort, or fatigue, and we note this as an important consideration for future interface refinement and evaluation.

The reason why we choose upper body as the haptic feedback area is based on following considerations:

Spatial coverage for directional discrimination: wind changes in this study spanned all principal compass directions. The encoding scheme decomposes arbitrary wind vectors into orthogonal components mapped to the front, back, left, and right panels of the suit. A waist belt or single-shoulder device would substantially reduce the available spatial resolution: a belt provides a horizontal ring of feedback but no clear front–back or elevation differentiation from the operators’s perspective, while a shoulder device restricts feedback to a single location. Full upper-body coverage was therefore necessary to preserve the directional resolution that the task demanded.

Postural stability of the torso: during VR supervisory control, the head rotates frequently as operators look at different displays, and the arms shift position during controller operation. The torso, by contrast, maintains a relatively stable orientation relative to the operator’s frame of reference throughout the session, making it the most reliable anchor for spatially coded directional cues.

Avoiding interference with occupied body segments: the head was occupied by the VR headset, and the hands and forearms were used for controller operation. These constraints ruled out head-mounted or hand/arm-worn haptic devices, leaving the torso as the natural candidate.

Physical burden: the bHaptics suit is lightweight and flexible; participants completed all experimental sessions without reporting issues that required stopping or modifying the haptic condition. We acknowledge, however, that prolonged continuous vibration in longer deployments may introduce adaptation or fatigue concerns, which we retain as a noted limitation.

When combined with the traditional visual panel in the user interface, the haptic augmentation system creates a comprehensive and immersive perceptual experience. The visual panel accurately presents wind direction and speed, while the haptic suit simultaneously provides a physical sensation, enabling operators to feel environmental changes in real time. This dual-modality design is intended to support situational awareness by distributing wind cues across visual and tactile channels, potentially reducing the need for continuous visual scanning. However, tactile decoding demands may also increase cognitive load depending on mapping complexity. Continuous haptic awareness of wind helps timely trajectory corrections, enabling safer and more efficient navigation in rapidly changing urban environments and supporting rapid, well-informed decision-making.

### 3.5 User interface module

**FIGURE 3:**
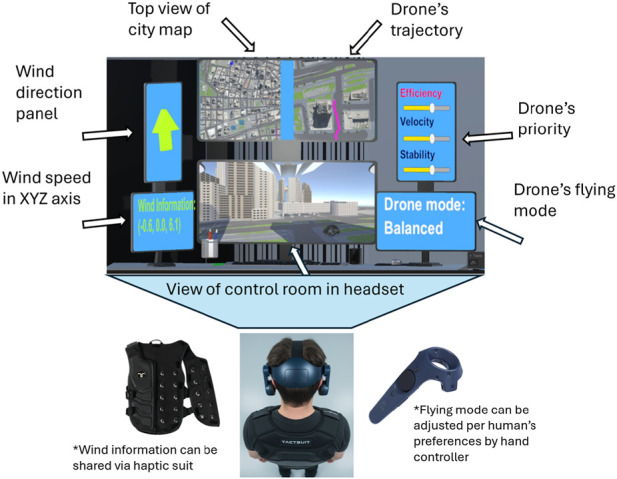
Human-subject experiment interface design.

The user interface (UI) module displays real-time drone imagery and sensor data on traditional multi-monitor workstations. This module also enables operators to pause, override, or modify the flight patterns of AI-controlled drones when necessary. Figure 3 illustrates the user interface within the simulator. As shown, three sets of dual-screen displays are arranged on the control console. The left dual screens aggregate wind field information: wind direction is displayed via large arrows whose colors indicate wind strength, while wind speeds along the X, Y, and Z-axes are listed in the wind condition information panel below. This layout enables operators to quickly grasp wind direction trends and confirm specific wind speed parameters through numerical values.

Upper screen in the middle is a top view city map displaying the drone’s planned and executed flight trajectory, enabling participants to observe the drone’s route in real time. Main screen below is a first-person view video that streams the drone’s forward field of vision, providing street-level environmental information. The control panel on the right displays priority settings for the drone’s current environment, featuring three sliders for efficiency, speed, and stability. Moving these sliders will switch between three autonomous modes: aggressive, balanced, and cautious. Alternatively, users can toggle modes directly via buttons. The three sliders express high-level priority weights that the drone’s path-planning module translates into one of three operational modes: aggressive, balanced, or cautious. In concrete operational terms, the mode determines the drone’s minimum safety distance threshold relative to obstacles during path planning. Aggressive mode sets the shortest buffer distance (prioritizing mission speed), cautious mode sets the largest buffer distance (prioritizing obstacle clearance margin), and balanced mode uses an intermediate value. The sliders therefore allow operators to express a preference for mission pace versus safety margin without requiring detailed knowledge of the underlying planner.

Beyond the desktop, the same layout is mirrored inside a VR headset display, preserving the control-room arrangement and human-first control hierarchy. Wind information is presented through two channels: on-screen numbers and arrows, and a haptic vest that maps wind direction to body-localized cues while scaling vibration with wind strength. A handheld controller supports quick mode changes and fine adjustments of priorities in VR. The system records slider movements, manual overrides, and timestamps for major events such as trajectory replans. These logs are used to analyze how participants balance efficiency and safety, how quickly they react to environmental changes, and which modalities support reliable decisions. Furthermore, this research allow human subjects to report changes in wind field perception through any sensory channel. Specifically, subjects use the controller to report shifts in wind direction and speed, serving as markers for their “belief state updates.” Critical data including timestamps, reported wind direction changes, reported wind speed changes, and the drone’s status during wind speed reports was recorded.

## 4 Experiment and methods

### 4.1 Participants

**FIGURE 4:**
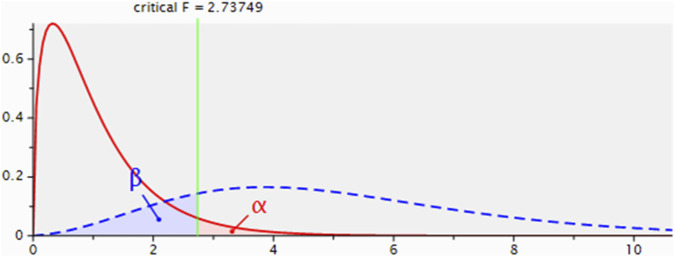
Sample-size justification (power = 0.90).

To explore the practical effects of the hypothesized belief updating system, we recruited 30 participants for a human experiment. *A priori* power analysis was conducted in G*Power (v3.1.9.7) for a repeated-measures ANOVA with one within-subjects factor shown in Figure 4. This study specified a medium effect size f=0.25 , two-tailed α=0.05 , desired power 1−β=0.90 , correlation among repeated measures is 0.50, and non-sphericity correction ε=1 . Under these settings, G*Power indicated a minimum total sample size of N=30 to achieve the target power (output: critical F=2.7094 , numerator df=3 , denominator df=87 , non-centrality parameter λ=15.00 , actual power =0.9037 ). Therefore 30 participants were recruited, which provides at least 0.90 power to detect an effect of f=0.25 in this design. Each participant received a $30 Amazon gift card as compensation, was approved by the University of Florida Ethics Committee (IRB202400072). All participants provided written informed consent before participating in the experiment.

Table 3 shows the demographic information of the subjects. The selection process was intentionally multidisciplinary, involving students from engineering, computer science, and psychology. This diverse selection was intended to assess how users with varying levels of familiarity with technology and robotics would perform when using the belief update collection system we developed. Most participants had limited experience with VR and 3D gaming and little experience with tasks such as controlling drones. All participants were right-handed and reported no known motor impairments or neurological abnormalities. Standard demographics such as age and gender were collected for possible exploratory analysis. Although the study focused primarily on age or gender differences, these data can help understand differences in performance across subgroups that may be influenced by physical strength, cognitive ability, or prior experience.

**TABLE 3:** Demographic information of the participants.

Category		Number	Percentage
Gender	Male	22	73.33%
	Female	8	26.67%
Age group	18–24	8	26.67%
	25–30	21	70.00%
	31 and older	1	3.33%
Major	Engineering (civil, transportation, construction, and related)	18	60.00%
	Computer science, Electrical and computer engineering	7	23.33%
	Psychology	5	16.67%

### 4.2 Task procedure

**FIGURE 5:**
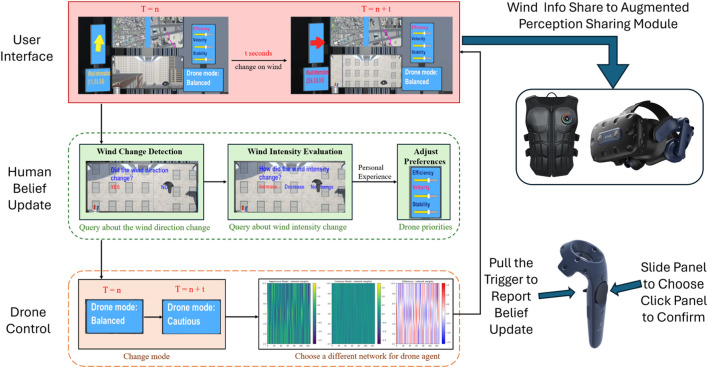
Human-subject experiment procedure.

To comprehensively evaluate how the proposed system influences belief state updates during human supervision of drone missions, this study designed an information acquisition and environmental assessment task using autonomous drones as shown in Figure 5. Each participant must immediately report any perceived changes in wind direction, then adjust their priorities and patterns based on their own judgment to adapt to the current situation whenever they deem adjustments necessary. The experiment employed a within-subject (repeated-measures) design with four conditions conducted in a simulation environment: control condition (information panel), augmented visual condition, haptic condition, and multimodal condition (augmented visual + haptic feedback). In the control condition, participants operated the drone within the VR environment for evaluation using only raw sensor data. Under the augmented visual condition, participants received both wind data via an information panel and enhanced vector graphics indicating wind characteristics. Under the haptic condition, the system translated wind information into upper-body haptic feedback via a haptic suit, omitting the information panel. The multimodal condition integrated all sensory inputs, providing participants with a wind direction panel, information panel, and haptic suit feedback to deliver a comprehensive sensory experience.

Prior to the experiment, participants signed informed consent forms and completed background questionnaires to collect data on age, gender, and VR experience. After completing paperwork, all participants underwent training in the virtual environment to familiarize themselves with the VR system and its interactions. Training included adapting to the VR headset and haptic suit, as well as learning to use VR controllers to operate the user interface within the simulated environment. During training, participants completed one practice simulation identical to the formal test in all aspects except the drone flight route. Experimental settings and content remained consistent across all conditions, and the order of the four conditions was randomized across participants to reduce potential order and learning effects. Although a full counterbalanced design was not implemented, randomization was used to distribute possible sequence effects across participants and reduce systematic bias associated with practice or fatigue.

The primary dependent variables, which are reaction latency and time-resolved local probability, were measured through dedicated wind-change report presses, which are operationally and conceptually independent of slider adjustments. Slider usage constitutes a secondary supervisory behavior (priority management) that runs in parallel with, but does not feed into, the belief-update reporting mechanism. Because the slider interface was identical across all four conditions, any between-condition differences in slider usage would themselves be a downstream consequence of the feedback modality (operators who perceive environmental changes more clearly may also adjust priorities differently), rather than a source of confounding in the belief-updating measurements. Furthermore, since all conditions used the same slider layout and the same task structure, mode switching cannot systematically inflate or deflate latency or local probability in a direction that would artificially favor one modality.

### 4.3 Measures and analysis

This study used both the VR devices and the unity functions to record eye track data, the drone’s real-time position, and the time the participant made a belief state update. Task performance and human function were evaluated using belief state update accuracy, average delay, belief state update probability and eye track based cognitive load. Additionally, this study employed the NASA TLX questionnaire to evaluate participants’ self-reported cognitive load based on the MDMT questionnaire (Hart and Staveland, 1988).

#### 4.3.1 Latency and belief state update probability

This study quantify when a change in the environment is perceived and how reliably it is reported. Ground truth changing times $\left\{\tau_{k}\right\}$ were predefined and recorded by the simulator and recorded for each session. In implementation, wind-field simulation and Unity data logging are handled by two independent modules. Therefore, a redundant on-board data-collection module was implemented as a safety fallback to improve robustness to potential sampling-frequency mismatches. Wind changes follow an approximately periodic schedule $\tau_{k}=\varphi +kP$ with P≈20s was reconstructed, where the session phase $\varphi \in \left[0,P\right]$ was selected from a fixed 0.05 s grid by the same rules for all sessions. Let $\left\{t_{j}\right\}$ ​ be the set of click times, for each click. Because multiple wind changes can occur within a trial and participants may click multiple times, each click must be aligned to the most recent ground-truth change $\tau_{k}$ to avoid attributing responses to stale events; $\pi_{j}$ provides this alignment. Equation 4 shows the index of the most recent change:

$$\pi \left(j\right)=\max\left\{k:\tau_{k}\leq t_{j}\right\}.$$ (4)

Unless otherwise noted, the detection window is W=4s and includes both endpoints. As a robust check, this study optionally sweeps $W\in \left[0.5,6\right]s$ and verify that the hit rate saturates well below 3.8 s while the window-level false-alarm probability increases monotonically, confirming that W=4s is a conservative choice. A belief state update event (change $\tau_{k}$ ) is counted as detected if at least one click occurs within $\left[0,W\right]$ as shown in Equation 5:

$$B_{k}=1\left(\exists j:0\leq t_{j}-\tau_{k}\leq W\right).$$ (5)

For participant-indexed analyses, this study writes $B_{ik}$ for the same indicator. The Beta–Binomial model was introduced to estimate a condition-level detection probability for belief updating, rather than a time-resolved reliability curve. Specifically, the quantity of interest is the probability that a true wind-change event is reported at least once within the predefined detection window W under a given feedback condition. Because each wind-change event can be coded as either detected or not detected within W, the resulting data are naturally Bernoulli/Binomial at the event level, making the Beta–Binomial formulation a convenient Bayesian model for estimating condition-wise detection reliability with mild regularization. In contrast, the time-resolved reliability analysis is provided separately by the local-probability curve, which aggregates correctness labels in a moving temporal neighborhood to show how reporting reliability evolves over the course of a trial. For condition c , let $K_{c}$ donate the number of changes and $S_{c}=\sum B_{k}$ donate the number detected (Or $S_{c}={\sum_{i}\sum_{k}z}_{ik}$ and $K_{c}=\sum_{i}K_{ic}$ when aggregating over participants). A Beta prior is used because it is conjugated to the Binomial likelihood, yielding a closed-form posterior for $P_{c}$ . Jeffreys prior, $\text{Beta}\left(1/2,1/2\right)$ , is adopted as an objective default for a probability parameter, providing mild regularization without favoring any condition when sample sizes are limited. The probability that a change is reported within W seconds under condition c is estimated in a Bayesian Beta-Binomial model with a Jeffreys prior as Equation 6:

$$\left.P_{c}\right|S_{c},K_{c}∼\text{Beta}\left(\frac{1}{2}+S_{c},\frac{1}{2}+K_{c}-S_{c}\;\right).$$ (6)

For detected events, the latency is the gap between the change time and the earliest click in the window defined as Equation 7 and Equation 8. For detected changes ( $B_{k}=1$ ), latency is defined using the earliest report within the detection window because it best captures the first moment the operator’s belief update becomes externally observable. When $B_{k}=0$ , latency is undefined and is excluded from latency summaries to avoid conflating missed detections with delayed detections. To characterize how the belief state update probability changes over time, each human subject report is labeled correct if it falls within $\left[0,W\right]$ after the most recent change as Equation 9. Latency summarizes the first response to each change but does not describe how reliably reports are aligned with the most recent true change throughout a trial. Therefore, each report $t_{j}$ is labeled correct/incorrect using $y_{j}$ relative to the most recent ground-truth change $\tau_{\pi_{j}}$ . To obtain a time-resolved reliability curve, correctness labels are aggregated within a symmetric temporal neighborhood around time t , producing the local probability $\theta_{t}$ as shown in Equation 10 to Equation 12 which smooths moment-to-moment variability while preserving within-trial dynamics.

$$L_{k}=t_{j*}-\tau_{k},$$ (7)

$$j^{*}={\text{arg}}_{i:0\leq t_{j}-\tau_{k\leq W}}\min\left(t_{j}-\tau_{k}\right).$$ (8)

$$y_{j}=1\left(0\leq t_{j}-\tau_{\pi \left(j\right)}\leq W\right).$$ (9)

$$N_{t}=\sum_{j}1\left(t_{j}\in \left.\left(t-\frac{W}{2},t+\frac{W}{2}\right]\right)\right),$$ (10)

$$C_{t=}\sum_{j}1\left(t_{j}\in \left.\left(t-\frac{W}{2},t+\frac{W}{2}\right]\right)\right)y_{j},$$ (11)

$$\hat{\theta }\left(t\right)=\frac{C_{t}}{N_{t}}$$ (12)

**FIGURE 6:**
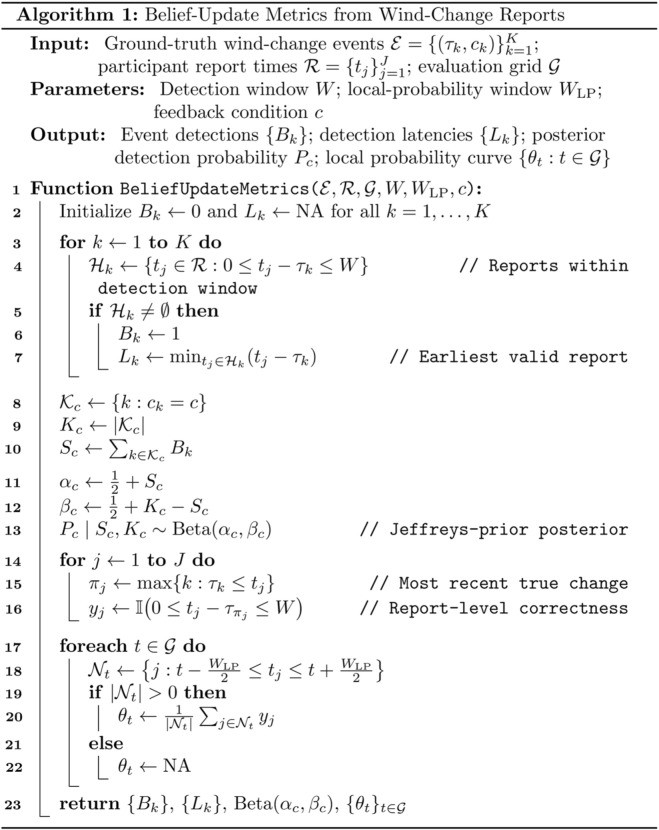
Pseudocode of belief update probability.

When $N_{t}=0$ , $\hat{\theta }\left(t\right)$ is undefined and omitted. Endpoints 0 and W are inclusive. Multiple clicks after the same change can each be correct for the local probability, but only the first correct click contributes to latency. The time-resolved local curve provides descriptive probability summaries over time. Latency statistics are descriptive unless otherwise specified. Ground-truth change times were recorded by the simulator and used directly. When a log was incomplete, an equally spaced schedule $\tau_{k}=\varphi +kP$ with a fixed 0.05 s phase grid was used as a uniform reconstruction rule, analyses using this rule led to the same substantive conclusions. Figure 6 shows the pseudocode of the overall algorithm of belief update probability.

#### 4.3.2 Objective cognitive load from eye-tracking data

This study constructed an objective eye-tracking index of cognitive load (Van Orden et al., 2001). The input streams were timestamp, three-dimensional gaze vector, right and left pupil diameters, luminance, and blink rate. Preprocessing removed missing values and outliers, linearly interpolated short gaps. For pupil component (Kahneman and Beatty, 1966; Beatty, 1982), this study averaged the two eyes pupil size as Equation 13 where pd is the diameter of pupil, then regressed out luminance using Equation 14 and Equation 15, and computed baseline-relative dilation as Equation 16. Here α and β are estimated within each participant using ordinary least squares, and $pd_{corr}\left(t\right)$ denotes the luminance-controlled pupil signal (the regression residual).

$${pd}_{avg}=\frac{1}{2}\left({pd}_{left}+{pd}_{right}\right),$$ (13)

$${pd}_{avg}\left(t\right)=\alpha +\beta lum\left(t\right)+\varepsilon \left(t\right),$$ (14)

$${pd}_{corr}\left(t\right)={pd}_{avg}\left(t\right)-\left(\alpha +\beta lum\left(t\right)\right),$$ (15)

$${pupil}_{\%}=100\cdot \frac{{pd}_{corr}-b}{b},$$ (16)

The baseline b is computed as the mean of $pd_{corr}\left(t\right)$ over an initial stable segment (or a running baseline window), so the dilation is expressed relative to the luminance-corrected baseline. For gazing component (Goldberg and Kotval, 1999), with unit gaze $g_{t}$ , we obtained angular velocity $w_{t}$ as Equation 17 and local dispersion $\sigma_{t}=std\left(g_{t⊥w}\right)$ , their sum indexes instability. For blink component (Stern et al., 1994), we computed a rolling blink rate $\rho_{t}$ and smoothed it. Each component was standardized within participant using a robust z-score shows in Equation 18. Then we can get the cognitive load for each subject by linearly fusing all three components equally using Equation 19 and Equation 20 to avoid outcome-dependent tuning and to preserve transparency and reproducibility:

$$w_{t}=\frac{\arccos\left(\langle g_{t},g_{t-1}\rangle \right)}{\Delta t},\;g_{t}=\frac{{\text{eyePoint}3D}_{t}}{{∥\text{eyePoint}3D}_{t}∥}$$ (17)

$$z\left(x\right)=\frac{x-median\left(x\right)}{IQR\left(x\right)},$$ (18)

$${cog}_{raw}=\omega_{p}z\left({pupil}_{\%}\right)+\omega_{g}z\left(w_{t}+\sigma_{t}\right)+\omega_{b}z\left(\rho_{t}\right),$$ (19)

$$\omega_{p}=\omega_{g}=\omega_{b}=\frac{1}{3}$$ (20)

**FIGURE 7:**
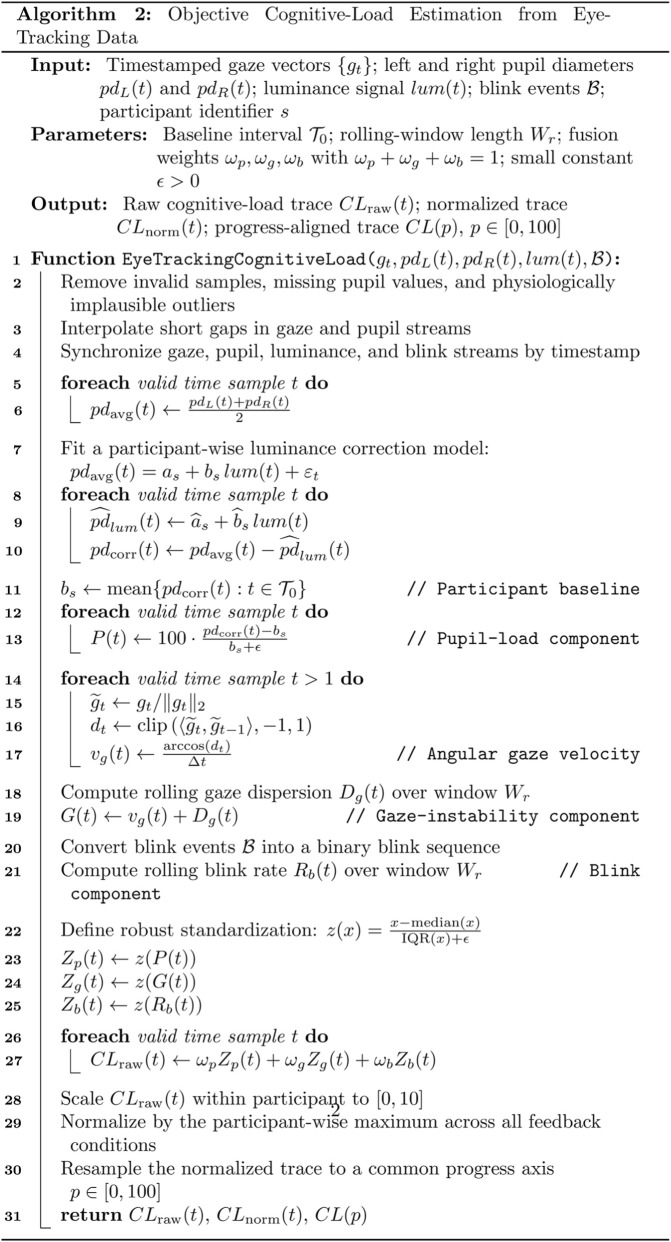
Pseudocode of objective cognitive load from eye tracking.

For between-condition comparability, values were first scaled within participant to $\left[0,10\right]$ , then normalized by the participant’s maximum across all four conditions, yielding the percent of subject max. Time was resampled to a common progress axis $p\in \left\{0,1,2,\ldots ,100\right\}$ to produce eye track data based on objective cognitive load. On the progress-aligned curves, we computed condition means with 95 percent confidence intervals. Omnibus differences were tested with the Friedman test and summarized by Kendall’s W . Pairwise comparisons used Wilcoxon signed-rank tests with Holm adjustment, and effect sizes were reported as $r=\left|Z\right|/\sqrt{N}$ . Figure 7 shows the pseudocode of the objective cognitive load from eye tracking algorithm.

## 5 Results

This section evaluates how feedback modality influences the speed and reliability of belief updating, and whether performance gains are accompanied by additional cognitive cost. Reaction time to environmental changes is first compared across conditions, followed by a time-resolved reliability analysis. Subjective workload is then assessed using NASA-TLX, and objective cognitive load is quantified from eye-tracking signals. The section concludes by examining whether performance and workload trends are consistent across these complementary measures.

### 5.1 Latency and belief update probability

**FIGURE 8:**
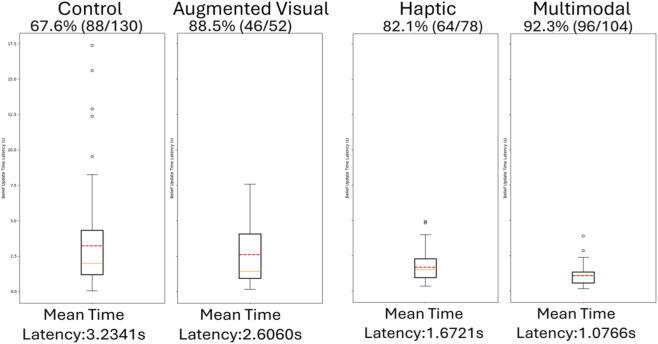
Reaction time latency.

Figure 8 summarizes reaction-time distributions for detecting wind changes under the four feedback conditions. In the Control condition (information panel), the mean reaction time was 3.23 s. The distribution included a long tail with several high outliers, indicating that responses were occasionally delayed when wind information was conveyed only through the XYZ vector values shown on the panel.

Augmented Visual feedback reduced the mean reaction time to 2.61 s, suggesting that visually augmented cues supported faster change detection than the panel-only baseline. Adding Haptic feedback further reduced the mean reaction time to 1.67 s. Compared with Augmented Visual, the haptic condition showed more discretized reaction-time values, which may reflect individual differences in sensitivity to vibrotactile cues and the corresponding decision threshold for reporting a change.

The Multimodal condition produced the fastest responses, with a mean reaction time of 1.08 s and a more concentrated distribution. This pattern indicates that combining augmented visual and haptic cues improved response speed while also reducing variability relative to single-modality feedback.

Table 4 reports the inferential statistics for reaction-time latency across conditions. Relative to the Control condition, only the Multimodal condition showed a statistically significant reduction in reaction time. In addition, the Haptic condition differed significantly from the Multimodal condition. All other pairwise comparisons were not statistically significant.

**TABLE 4:** Statistical results of the reaction time latency.

Condition	Control vs. visual	Visual vs. haptic	Haptic vs. multi	Control vs. haptic	Visual vs. multi	Control vs. multi
Reaction delay (s)	No difference (p = 0.086)	No difference (p = 0.388)	Smaller (p = 0.045)	No difference (p = 0.174)	No difference (p = 0.238)	Larger (p < 0.001)

**FIGURE 9:**
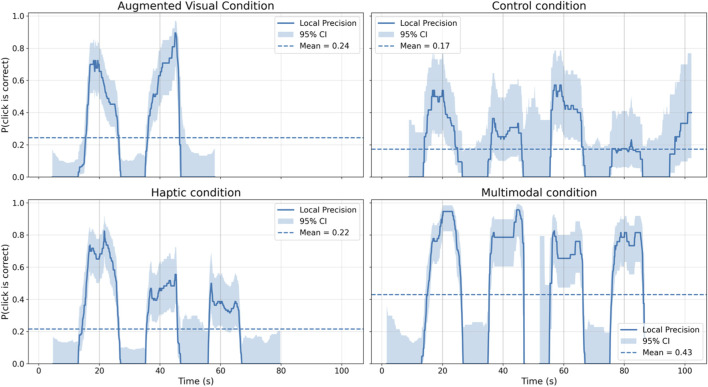
Local precision across conditions.

To evaluate reliability over time (beyond a single latency summary), Figure 9 plots the time-resolved probability that a reported change was correct within each wind-change interval. The solid blue curve shows the local probability with a 95% confidence interval, and the dashed line indicates the condition mean. The Control condition exhibited extended periods in a low-probability range and the lowest mean value (0.17). Augmented Visual feedback increased the mean to 0.24 and produced more frequent high-probability epochs that persisted longer across intervals. The Haptic condition yielded a similar mean (0.22) but showed shorter high-probability windows and larger variability, despite exhibiting early peaks. The Multimodal condition produced the highest mean (0.43) and sustained high-probability plateaus (approximately 0.7–0.9) across most change windows, with comparatively tight confidence bands.

Given the gradient of local probability across conditions, the Control condition almost certainly had the highest miss rate, meaning its reported latency of 3.23 s is already an optimistic figure. Any latency bias therefore runs in the direction of underestimating the Control–Multimodal performance gap, making our reported conclusions conservative rather than inflated.

Overall, these time-resolved curves suggest a graded improvement in reliability from Control to Multimodal feedback. While haptic-only feedback did not consistently sustain high local precision relative to augmented visual cues, combining visual and haptic feedback yielded the most reliable performance over time.

### 5.2 NASA task load index

**FIGURE 10:**
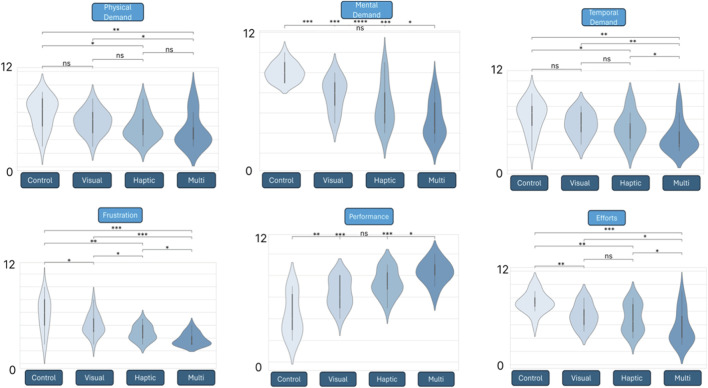
NASA task load index result.

NASA Task Load Index (NASA-TLX) was used to assess subjective workload and perceived performance across conditions. Figure 10 summarizes the six NASA-TLX dimensions, and the corresponding statistical results are reported in Table 5. Participants in the Control condition reported the highest mental and physical demand, whereas the Multimodal condition produced the lowest ratings on both dimensions. Temporal demand was also highest in the Control condition and differed significantly from other conditions.

**TABLE 5:** Results of the NASA TLX.

Condition	Mental demand	Physical demand	Temporal demand	Performance	Effort	Frustration
Control vs. visual	Larger (p < 0.001)	No difference (p = 0.080)	No difference (p = 0.105)	Larger (p = 0.004)	Larger (p = 0.005)	Larger (p = 0.047)
Visual vs. haptic	No difference (p = 0.094)	No difference (p = 0.156)	No difference (p = 0.412)	No difference (p = 0.070)	No difference (p = 0.540)	Larger (p = 0.021)
Haptic vs. multi	Larger (p = 0.049)	No difference (p = 0.197)	Larger (p = 0.036)	Larger (p = 0.029)	Larger (p = 0.032)	Larger (p = 0.022)
Control vs. haptic	Larger (p < 0.001)	Larger (p = 0.012)	No difference (p = 0.049)	Larger (p < 0.001)	Larger (p = 0.005)	Larger (p = 0.002)
Visual vs. multi	Larger (p < 0.001)	Larger (p = 0.039)	Larger (p = 0.005)	Larger (p < 0.001)	Larger (p = 0.015)	Larger (p < 0.001)
Control vs. multi	Larger (p < 0.001)	Larger (p = 0.005)	Larger (p = 0.001)	Larger (p < 0.001)	Larger (p < 0.001)	Larger (p < 0.001)

Perceived performance was rated highest in the Multimodal condition, indicating greater confidence in judgments, while the Control condition received lower performance ratings. Effort showed the same overall pattern: the Control condition required the greatest effort and the Multimodal condition required the least. Frustration ratings were consistent with these effort trends.

Across most dimensions, Table 5 indicates significant differences for multiple condition pairs, suggesting that feedback modality influenced subjective workload. In contrast, differences between the Augmented Visual and Haptic conditions were not significant for several dimensions, implying that each modality provided comparable subjective support when presented alone. However, combining the two modalities yielded the most favorable workload profile overall: the Multimodal condition generally produced the lowest workload ratings and the highest perceived performance, while the Control condition consistently exhibited the highest workload scores. Taken together, these results indicate that multimodal feedback was associated with reduced subjective workload and improved perceived performance relative to the information panel-only baseline.

### 5.3 Cognitive load from eye track data

To complement the subjective measures, eye tracking was used to derive an objective, time-varying indicator of cognitive load. The analysis used multiple recorded channels, including timestamps, the three-dimensional gaze vector, binocular pupil diameters, luminance, and blink rate. Missing samples and outliers were removed, and short gaps were linearly interpolated. To extract load-related variation, the left and right pupil signals were averaged and then corrected for baseline and luminance effects: the luminance-driven component was regressed out, and baseline-relative pupil dilation was computed from the residual signal. Gaze dynamics were quantified from frame-to-frame changes in the gaze vector to obtain motion speed and stability indices, and blink rate was computed as an additional indicator of momentary resource strain.

**FIGURE 11:**
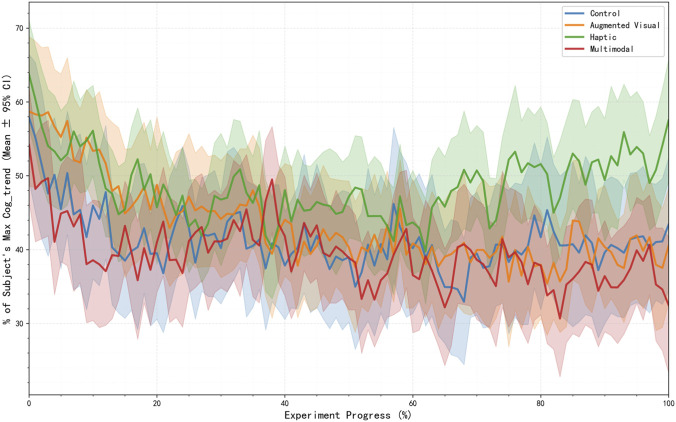
Objective cognitive load from eye track data.

These components were standardized within each participant, direction-aligned, and linearly fused into a single continuous cognitive-load index, where larger values indicate higher instantaneous load. The computation of the index was kept identical across conditions; only two harmonization steps were applied at the analysis stage to support between-condition comparison. First, a participant-wise percentage normalization divided the index by the participant’s maximum value across all four conditions, reducing between-subject scale differences. Second, the time axis was resampled to 0%–100% of experimental progress to align trajectories by progress and enable group-level means and confidence intervals. Figure 11 shows the resulting cognitive-load trajectories produced by this pipeline.

Across all conditions, cognitive load is highest at the beginning of the session and then drops sharply during the early portion of the task (approximately the first 10%–20% of progress), consistent with an initial adaptation period. After this early decline, the Multimodal condition remains comparatively lower and more stable for most of the session. The Control and Augmented Visual conditions track in a similar mid-range band with overlapping confidence intervals, suggesting broadly comparable objective load levels across much of the task. In contrast, the Haptic condition shows a higher trajectory in the second half of the session and a tendency to increase toward the end, accompanied by wider confidence bands, indicating greater between-participant variability in objective load under haptic-only feedback.

**FIGURE 12:**
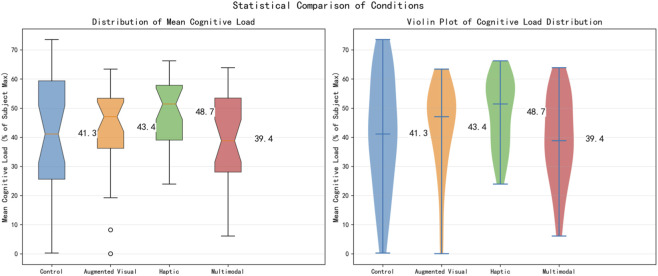
Cognitive load statistical comparison of different conditions.

Figure 12 and Table 6 summarize the eye-tracking–derived cognitive-load distributions across conditions, expressed as a percentage of each participant’s within-subject maximum. The notched boxplots (left) and violin plots (right) report both central tendency (with medians/means annotated) and dispersion. The Haptic distribution is shifted upward relative to the other conditions, whereas Control and Augmented Visual cluster lower. The Multimodal distribution is comparable to, and in some cases slightly lower than, Control and Augmented Visual.

**TABLE 6:** Statistical results of the cognitive load based on eye track data.

Condition	Control vs. visual	Visual vs. haptic	Haptic vs. multi	Control vs. haptic	Visual vs. multi	Control vs. multi
Cognitive load	No difference (p = 0.9636)	Smaller (p = 0.0201)	Larger (p = 0.0026)	Smaller (p = 0.0191)	No difference (p = 0.1109)	No difference (p = 0.3811)

The pairwise nonparametric comparisons in Table 6 support these visual patterns. Cognitive load was significantly higher in the Haptic condition than in Augmented Visual (p = 0.0201), Control (p = 0.0191), and Multimodal (p = 0.0026). In contrast, Control and Augmented Visual did not differ (p = 0.9636), and neither Augmented Visual vs. Multimodal nor Control vs. Multimodal reached significance (p = 0.1109 and p = 0.3811, respectively). Taken together, these results indicate that haptic-only feedback elicited the greatest objective cognitive load, whereas the Multimodal condition did not show this elevation and remained statistically indistinguishable from Control and Augmented Visual.

Across conditions, reaction-time latency decreased monotonically from Control to Augmented Visual, then to Haptic, and finally to Multimodal. In contrast, the time-resolved correctness measure showed a small decrease in the Haptic condition relative to Augmented Visual. This divergence suggests that faster detection did not necessarily translate into more reliable belief updating under haptic-only feedback. Given that eye-tracking results indicate higher objective load in the Haptic condition, the next analysis examines whether elevated cognitive load can account for the reduced local probability observed in that condition.

**FIGURE 13:**
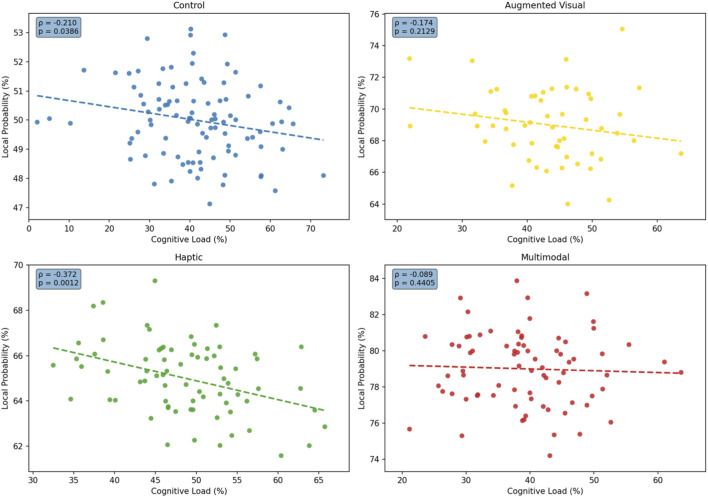
Cognitive load statistical comparison of different conditions.

Figure 13 tests whether the reduction in time-resolved correctness under haptic-only feedback is associated with elevated objective cognitive load. Local probability values were extracted from the time-resolved curves in Figure 9 and paired with the eye-tracking–derived cognitive-load value at the same experiment-progress percentile. Spearman’s rank correlation was then computed for each condition to assess monotonic associations without assuming a linear relationship.

Across conditions, the association between cognitive load and local probability was generally negative. The strongest and statistically significant correlation was observed in the Haptic condition (ρ = −0.372, p = 0.0012), indicating that higher objective load tended to coincide with lower local probability. The Control condition showed a smaller but significant negative association (ρ = −0.210, p = 0.0386). In contrast, the Augmented Visual condition (ρ = −0.174, p = 0.2129) and the Multimodal condition (ρ = −0.089, p = 0.4405) did not reach statistical significance.

These results provide converging evidence for the pattern in Figure 9: although haptic-only feedback reduced reaction-time latency, it did not sustain reliability to the same extent as augmented visual feedback. Figure 13 further illustrates that the Haptic condition exhibits the clearest negative association between load and local probability, consistent with a load-related performance cost under haptic-only cues. By comparison, the Multimodal condition combines cues without showing a significant load–reliability trade-off, which is consistent with the interpretation that multimodal feedback mitigates the cognitive cost associated with haptic cues alone. Finally, these findings should be interpreted as correlational rather than causal; nevertheless, they support the hypothesis that elevated cognitive load is a plausible mechanism contributing to the reduced local probability observed under haptic-only feedback, despite faster detection overall.

## 6 Discussion

The key finding of this study is that the utility of an interface integrating multiple sensory modalities is not merely additive, but rather synergistic, fundamentally enhancing a human operator’s ability to form and update belief states about a dynamic environment while supervising an autonomous drone. The results provide clear empirical evidence for this claim. Across all key performance indicators, the multimodal condition which combined augmented visual and haptic feedback demonstrated superiority compared to other conditions. It yielded the fastest mean reaction latency at 1.08 s, significantly outperforming the control condition and the haptic conditions. It also produced the highest mean local probability of a correct update, sustaining high-precision plateaus far more consistently than the control, augmented visual, or haptic conditions. Finally, subjective workload, as measured by NASA-TLX, was lowest for the multimodal condition across all six dimensions, with most differences being statistically significant. These performance gains reveal a deeper cognitive mechanism: the multimodal system is not just better in optimizing data, but in aligning the presentation of complex, dynamic information with the natural architecture of human sensory processing.

As established in the introduction, a core challenge in human-drone interaction is cognitive overload, where the pace and complexity of information exceed human processing capacity. While unimodal feedback offers improvements, each has its limits. The multimodal system resolves this by integrating cues across different modalities. The haptic channel provides an immediate, embodied sensation that heightens the operator’s sensitivity to change, reflexively guiding their attention. Subsequently, the augmented visual channel delivers rich and direct context at that guided focal point for precise evaluation. This transforms the task from processing two isolated data streams into perceiving a single, coherent event, creating a “cognitive resonance” that makes the belief state updating process more fluid and less resource intensive. This represents a more profound human-drone alignment that occurs at the level of perception, belief and cognition.

To fully find out the source of the benefits of multimodal, it is essential to deconstruct the effect of the unimodal augmented visual and haptic feedback conditions. Augmented visual feedback’s primary strength lies in providing rich, direct context that is integrated with the environment using graphic information, it supports a more analytical and deliberate mode of belief updating. This information richness, however, comes at the cost of reaction speed. The augmented visual condition’s mean reaction time of 2.61 s, while an improvement over the control condition (3.23 s), was significantly slower than the haptic condition (1.67 s). This suggests that interpreting rich visual information requires focused attention and longer cognitive processing. Yet, this deliberate assessment yielded greater precision, with the Visual condition’s mean local probability of a correct update (0.24) exceeding that of the Haptic condition (0.22). This paints a picture of a reliable but relatively slow system that provides high quality information for considered judgment but lacks the capacity for immediate response. In contrast, haptic feedback fosters a powerful, ambient, and heightened sensitivity to environmental changes. It bypasses the crowded visual channel to trigger a rapid, embodied reaction, a fact strongly supported by its mean reaction time of 1.67 s, which is the fastest of any unimodal condition. This immediacy is invaluable in high stakes scenarios requiring swift responses.

However, this speed is accompanied by a significant and hidden cognitive cost. Objective eye-tracking data revealed that the haptic condition induced the highest cognitive load of all conditions, significantly greater than the augmented visual (p = 0.0201), Control (p = 0.0191), and Multimodal (p = 0.0026) conditions. This high load was directly linked to performance degradation, and a significant negative correlation was found between cognitive load and the local probability of a correct belief update in the Haptic condition (ρ = −0.372, p = 0.0012). The root cause is that abstract tactile signals, such as vibration patterns, lack inherent semantics. Operators must expend considerable cognitive resources to decode these signals and map them to specific environmental states. This finding exposes a potential “illusion of efficiency” in interface design, where reliance on reaction speed or subjective reports alone can be misleading. Objectively, operators in the Haptic condition were in a state of high cognitive strain and error-proneness, trading mental effort for lower decision accuracy without being consciously aware of it.

This deconstruction provides the foundation for understanding the multimodal condition’s success. Its synergistic effect stems from its masterful use of “cognitive offloading” and “cross-modal facilitation,” which effectively allocates the information processing burden and resolves the interpretive ambiguity of purely haptic feedback. The mechanism is one of mutual reinforcement: the haptic signal acts as an immediate sensory cue, making the operator sensitive to the location of an environmental change without requiring an active visual search. The augmented visual system, with its attention already directed, then instantly receives the explicit, semantic context from the augmented visual display, effortlessly clarifying the meaning of the haptic sensation. This process effectively eliminates the cognitively expensive decoding effort required in the pure haptic condition. The augmented visual display provides an instant interpretation for the haptic cue, and the haptic cue provides a search focus for the visual system.

The objective cognitive-load results suggest that the multimodal advantage is better explained by complementary information across modalities than by the task being too easy. Specifically, the haptic-only condition likely imposed the highest load because participants had to decode abstract vibration patterns and translate them into wind meaning without a concurrent semantic display, which increases interpretive effort even though detection can be fast. In the multimodal condition, this decoding burden was reduced because the two channels played complementary roles: haptic cues acted as rapid attentional signals, while the visual display provided explicit contextual interpretation of direction and magnitude. In other words, the operator did not need to rely on haptic information alone to infer the environmental state. This cross-modal complementarity offers a plausible explanation for why multimodal feedback maintained rapid responses while avoiding the elevated cognitive load observed under haptic-only feedback. Although the present task was structured and controlled, the combination of faster detection, higher time-resolved correctness, and lower objective load suggests that the benefit of multimodal feedback was not simply due to low task difficulty, but to more efficient cue integration under uncertainty. No formal qualitative interview data were collected; therefore, this interpretation should be viewed as theoretically grounded rather than directly self-reported by participants.

The findings of this study carry significant implications for both cognitive science theory and the practical design of safety-critical systems. They provide empirical evidence that challenges and extends classic models of belief updating, such as the Bayesian framework, which often assumes rational agent processing evidence independent of its presentation format. This study demonstrates that the sensory modality of the evidence is itself a critical variable that can dramatically alter the efficiency and accuracy of the updating process. A well-designed multimodal interface can act as a “cognitive support,” helping human operators perform closer to a normative ideal by mitigating the effects of processing limitations and cognitive biases. From these results, we can distill a set of actionable design principles for next-generation interfaces. For example, prioritizing sensory synergy, ensuring that channels are complementary and mutually reinforcing, using haptics to enhance sensitivity and direct attention, but always pair it with an unambiguous channel for interpretation to avoid high decoding costs, recommending the measurement of objective workload using physiological tools like eye-tracking to uncover hidden cognitive costs, and designing the system for context, not just for data, by embedding information within its operational environment as demonstrated by the success of augmented visual feedback. These principles have direct relevance for real-world applications such as search and rescue, logistics, surveillance, aviation, medicine, and process control.

It is necessary, however, to acknowledge the limitations of this work to ensure rigorous interpretation and guide future research. The study was conducted in a high-fidelity simulator that, despite its realism, cannot fully replicate all the stressors and sensory inputs of real-world operations. Furthermore, participants were primarily novices, the cognitive costs and benefits of these interfaces may differ for experienced professionals who have internalized specific information processing strategies. These limitations open several avenues for future inquiry. First is the development of adaptive interfaces that can dynamically adjust feedback strategies based on real-time, objective measures of an operator’s cognitive load. Second, longitudinal studies are needed to investigate how the cognitive costs and performance advantages of multimodal interfaces evolve with training and expertise. Third, *in-situ* field studies with professional drone operators are required to validate the robustness of these findings in authentic operational environments. Finally, the potential of other sensory modalities, such as 3D spatialized audio, could be explored as another channel for cognitive offloading and cross-modal facilitation.

An additional limitation is that the present design does not fully isolate all possible modality combinations. In particular, the study did not include a “control + haptic” condition that would combine the baseline information panel with haptic feedback alone. Such a condition would be valuable for determining whether the observed multimodal advantage arises primarily from redundancy, true cross-modal complementarity, or the specific contribution of augmented visual cues. Based on the current results, we expect a control + haptic condition to improve change-detection speed relative to the control condition, but to remain less effective than the full multimodal condition because the augmented visual channel appears to provide semantic support that helps reduce the decoding burden associated with haptic-only cues. More broadly, although this study focuses on localized wind estimation in urban drone supervision, the underlying framework is applicable to other supervisory tasks in which humans must update beliefs about partially observable and time-varying states from imperfect cues, such as obstacle-risk assessment in navigation, contact-state or force estimation in robotic manipulation, and mode monitoring in shared-autonomy systems. At the same time, the present implementation benefits from relatively structured environmental changes, including approximately periodic wind updates, which simplify event alignment and time-resolved analysis. In domains where uncertainty is more irregular, weakly segmented, or nonstationary, belief-update events may be harder to define and may require more flexible event-detection, continuous-state inference, or probabilistic segmentation methods. Future work should therefore include both additional modality ablations and broader task settings to more precisely disentangle the contribution of each feedback channel and to evaluate how well the framework transfers to less structured supervisory environments.

## 7 Conclusion

This study concludes that the synergistic integration of complementary sensory modalities is a practical design principle for enhancing human supervisory control of autonomous systems. Through a detailed analysis of augmented visual and haptic feedback, findings show that a multimodal interface dramatically improves an operator’s speed and accuracy in perceiving and updating beliefs about dynamic environmental conditions, far exceeding the performance of any single modality used in isolation. The fundamental reason for this advantage is that a well-designed multimodal system aligns with the basic architecture of human perception. It leverages one sense (haptic) to provide immediate, heightened sensitivity that directs attention, while simultaneously using another sense (visual) to provide instant, contextual interpretation, thereby facilitating fluent cognitive processing. This approach transcends merely presenting more data to an operator; it structures information in a cognitively economical way that effectively mitigates overload.

In an age of increasing automation and data complexity, where human cognitive capacity is often the bottleneck in system performance, findings illuminate a path forward. The goal of interface design must shift from creating mere “data pipelines” to engineering true “cognitive partners” for the human operator. This is critical for ensuring the safety and effectiveness of high-stakes applications, from emergency response to urban logistics. This work represents a foundational step toward achieving a deeper human-machine symbiosis. The objective should not be merely to prevent error, but to create human robot teams that are more resilient, perceptive, and capable than either human or machine alone. By designing interfaces that work in seamless concert with human sensory and cognitive processes, we can unlock the full potential of human-autonomy teaming to build smarter, safer, and more effective strategies for tackling the complex challenges in real world.

## Data Availability

The raw data supporting the conclusions of this article will be made available by the authors, without undue reservation.
